# A Case of Ossified Tissue Misdiagnosed as Amyloid Deposits by Congo Red Stain and Birefringence

**DOI:** 10.7759/cureus.75179

**Published:** 2024-12-05

**Authors:** Hajime Iwata, Jotaro Nakashima, Tomonori Kai, Haruka Okada

**Affiliations:** 1 Pathology, Tokyo Metropolitan Tama Medical Center, Fuchu, JPN

**Keywords:** amyloid, amyloid deposits, birefringence, bone ossification, collagen autofluorescence, congo red stain, pulmonary ossification

## Abstract

Pulmonary amyloidosis is diagnosed by identifying amyloid deposits using Congo red stain (CR) and birefringence under polarized light. However, collagen fibers can also produce similar staining results, complicating diagnosis. We report a case of a 55-year-old male patient with lung opacities, initially suspected to have amyloidosis based on CR positivity and green birefringence. Thoracoscopic biopsy revealed pulmonary ossification (PO) rather than amyloidosis. The parallel alignment of lamellar bone fibers likely caused false-positive CR findings and birefringence. This case underscores the need for careful differentiation between bone tissue and amyloid deposits. Misdiagnosis can lead to unnecessary treatments, especially when the actual condition requires only conservative management, as in PO. Awareness of PO as a mimic of amyloidosis is crucial, especially in cases lacking other systemic symptoms or calcification.

## Introduction

Amyloidosis is a group of diseases characterized by amyloid fibril deposition in various organs and tissues. The deposits may be local, as in pulmonary amyloidosis, or systemic i.e., occurring in multiple organs. In humans, 42 proteins have been identified as amyloidogenic [1]. Regardless of the type of the precursor protein, amyloid fibrils have a uniform ultrastructure. They can be detected using Congo red (CR) stain, direct fast scarlet (DFS) stain, or polarized light microscopy [2]. While these examinations are essential to diagnose amyloidosis, the results are not specific to amyloid proteins because collagen fibers also produce similar findings on these examinations [3,4]. Accurate diagnosis of amyloidosis is vital, as misdiagnosis can lead to unnecessary treatments. To date, no studies have specifically reported that bone tissue exhibits properties similar to amyloid deposits. We report the first case in which a patient’s ossified tissue was mistakenly interpreted as amyloid deposits. His chest X-ray demonstrated increasing lung opacities which were later identified as pulmonary ossification (PO). The background characteristics that can help prevent a misdiagnosis are also discussed. This case report was previously presented as a poster at the 68th Autumn Special Meeting of the Japanese Society of Pathology on November 17, 2022.

## Case presentation

**Figure 1:**
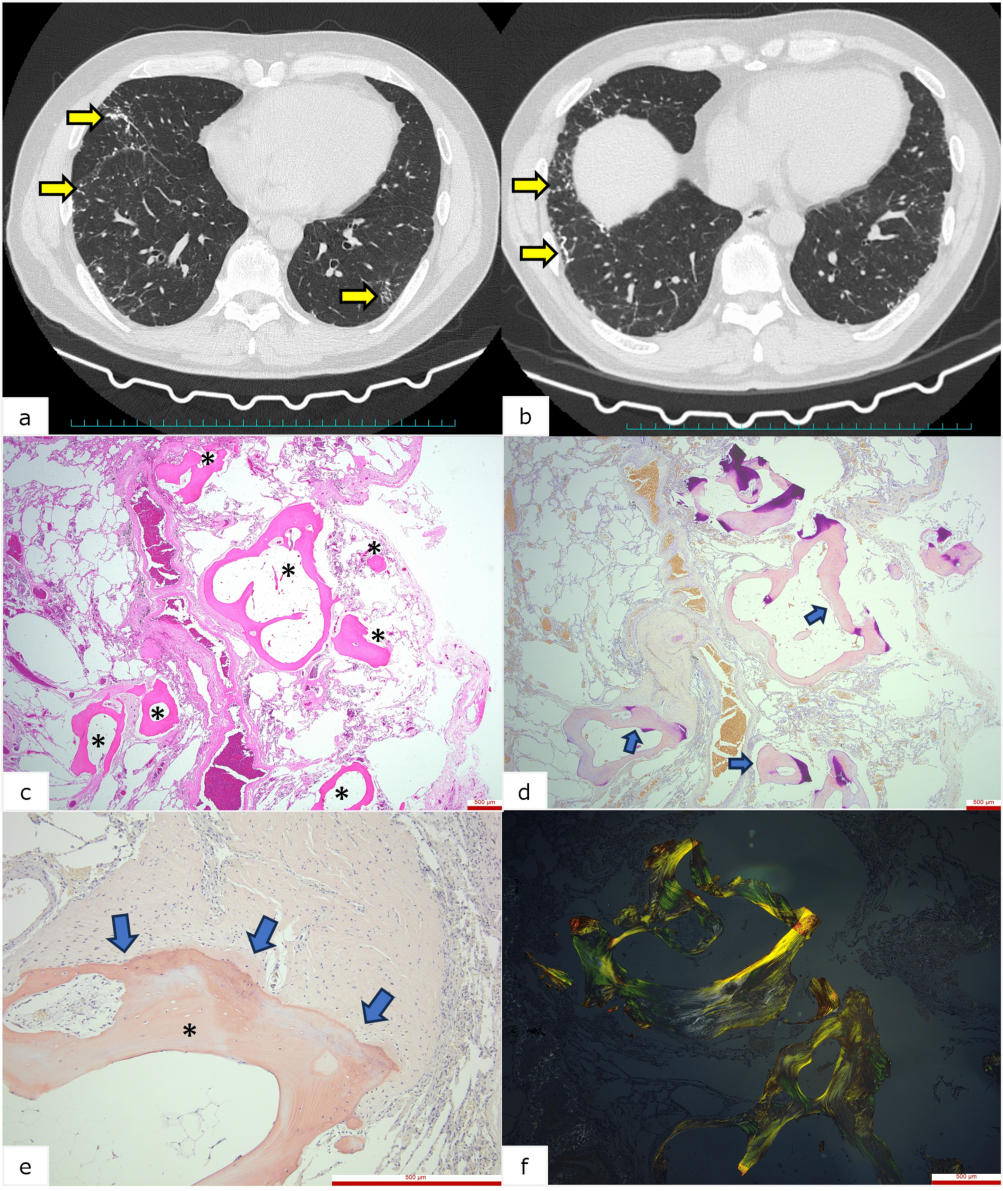
Radiologic and histopathological features of diffuse pulmonary ossification (a) Mediastinum-level computed tomography (CT) findings. (b) Diaphragm-level CT findings. Both (a) and (b) show diffuse, high-attenuation, micronodular opacities in all lung fields (yellow arrows). (c) Hematoxylin and eosin stain reveals mild fibrosis and bone in the alveoli and nearby vessels (asterisks). (d) Congo red (CR) stain demonstrates orange-red staining of bone tissue near vessels and the alveolar septa, with some parts of the bone matrix are amorphous (blue arrows). (e) Direct fast scarlet (DFS) stain highlights bone (asterisk) and shows surrounding fibroblasts and collagen fiber proliferation, with a transition zone from fibroblasts to bone tissue (blue arrows). (f) The green-brown birefringence of bone tissue under polarized light microscopy.

A 55-year-old male patient with a history of hypertension, hyperuricemia, and post-tonsillectomy IgA nephropathy was referred to the hospital for increasing lung opacities (Figure 1a, 1b). He had used a down feather duvet for over ten years and had no history of smoking or asbestos exposure. When blood tests found no abnormalities, a thoracoscopic biopsy was performed. A lung biopsy demonstrated scattered bone in the alveoli. Thickened septa and minimal, perivascular inflammation were observed around the bones (Figure 1c). While CR and DFS were positive within the bones (Figure 1d, 1e), and green-brown birefringence was clearly visible under polarized light microscopy (Figure 1f), there was no evidence of amyloid deposits. Amyloidosis of the diffuse alveolar-septal type was tentatively diagnosed, and immunostaining for amyloid A, kappa, lambda, beta two-microglobulin, and transthyretin was performed to ascertain the type of amyloidosis. All the immunological results were negative, leading to the conclusion that the collagen fibers in the bone might have been responsible for the earlier, positive findings.

## Discussion

Collagen fibers are known to produce a false positive finding on CR and green birefringence under polarized light microscopy [3]. Any tissue component can bind to CR molecules in an orderly manner. In the present case, the tissue was composed of lamellar bone, which runs along the long axis and resembles the trabeculae of cancellous bone (Figure 1c). This parallel structure could have been the cause of the false positive findings and birefringence although the mechanism by which it may produce these findings is not fully understood. CR molecules typically bind to amyloid deposits via their beta-pleated, sheet structure, which involves hydrogen bonding and a hydrophobic interaction between the CR molecules and the amyloid proteins [5]. This connection forces the CR molecules into highly oriented, linear, parallel arrays which also help create the green birefringence associated with amyloid fibrils under polarized light. Thus, in the present case, instead of binding to the beta-pleated sheet structure, CR was bound to the lamellar structure of the bone. This was likely to have caused the false positive findings.

In addition to the structural factor, attention must also be paid to color variation. Although amyloid is generally observed as apple green under polarized light, other colors such as yellow, blue, orange, and red can also be seen [6]. Furthermore, the collagen fiber component is likely to have contributed to the false positive results. Collagen exhibits autofluorescence, which resembles birefringence and occurs when type I and type III collagens are exposed to an excitation light source. Pyridinoline, a crosslinking compound, is a native fluorophore occurring in the dermis and produces a yellow autofluorescence [3], which can be easily confused with the birefringence of amyloid deposits.

Understanding collagen and bone formation helps elucidate the PO process. Bones contain collagen fibers because osteoblasts produce collagen and osteocalcin, which form the non-calcified bone matrix. They also release matrix vesicles rich in alkaline phosphatase and acidic phospholipids, which promote hydroxyapatite crystallization. These crystals bind to the collagen fibers to calcify the osteoid [7]. Through this process, osteoblasts integrate collagen fibers into bone formation. In PO, chronic inflammation contributes to fibrosis, transforming fibroblasts into osteoblasts. This change, critical to the pathomechanism of PO, is also influenced by increased shear stress, as seen in pulmonary fibrosis and chronic injuries [8]. Thus, PO involves tissue injury resulting from a variety of causes. In the present case, the transition from fibroblasts to osteoblasts and osteocytes was observed near the bone tissues, which identifies the tissues as bone rather than amyloid (Figure 1e).

Identifying the causes of PO is crucial for accurately diagnosing and managing this condition. However, the precise cause of PO is unknown, though in general, the condition is thought to be idiopathic or related to a pulmonary disorder, cardiac disorder, or an extra-cardiopulmonary disorder [8]. The present case might be classified as idiopathic due to the absence of any other obvious etiology. A dendriform pattern is commonly associated with idiopathic PO. Although this pattern often stems from interstitial pneumonia, this was not the case in the present patient. The morphological features of PO and amyloidosis are different; while amyloid appears as glassy amorphous eosinophilic material, PO is characterized by organized, lamellar bone or spicules with varying levels of ossification and occasional marrow elements [8].

The misdiagnosis of idiopathic PO as amyloidosis affects the treatment strategy. In the present case, the dendriform pattern was also misleading because it resembled diffuse alveolar-septal amyloidosis, which is generally a manifestation of systemic primary or light-chain (AL) amyloidosis, in which excess immunoglobulins are produced by clonal or malignant plasma cells. Its treatment reduces the concentration of circulating free light chains [2]. On the other hand, there is no proven treatment for idiopathic PO, which requires a more conservative approach [8]. Therefore, a misdiagnosis can certainly lead to unnecessary treatment. 

Detecting calcification is helpful for diagnosing amyloidosis because calcification is more common than isolated ossification in this disease. Secondary calcification can occur in amyloidosis because the amyloid fibrils have an affinity for calcium [8]. In amyloidosis, calcification is more commonly observed than isolated ossification due to the amyloid fibrils' natural affinity for calcium. While osseous metaplasia can develop as a secondary process following extensive tissue calcification and injury [8,9], ossification without calcification in amyloidosis is rare. In the present case, bony formations existed near the septa and vessels (Figure 1c-1e). However, in this case, amyloidosis was ruled out upon retrospective review because neither eosinophilic, amorphous amyloid deposits nor calcifications were found. 

Accurate diagnosis and subtyping of amyloidosis are essential for better outcomes. CR staining is a key method for amyloid detection, while immunohistochemistry helps identify serum amyloid A (AA), transthyretin (ATTR), and AL amyloid. In AL amyloidosis, mass spectrometry is necessary for fibril typing, especially when immunological tests are unclear. Early detection is crucial, as a delay can worsen cardiac health and increase transplant-related mortality. High-dose melphalan with autologous stem cell transplantation (HDM-ASCT) is effective for eligible patients, with one study showing a five-year survival of 86% for those achieving complete hematologic response [10]. These techniques help in early diagnosis and treatment, improving prognosis.

## Conclusions

In conclusion, we reported a rare case where PO was initially misdiagnosed as amyloidosis due to false positive CR staining and green birefringence. Recognizing the presence of bone tissue rather than amyloid deposits is crucial to prevent such diagnostic errors. In challenging cases, additional immunostaining or even mass spectrometry may be necessary to confirm the diagnosis accurately. This approach not only avoids misdiagnosis but also ensures appropriate management, ultimately improving patient outcomes. Further studies are needed to refine diagnostic criteria and enhance our understanding of conditions like PO.
